# Effect of Exogenous Melatonin Supply on Potato Plants Grown In Vitro

**DOI:** 10.3390/antiox14080917

**Published:** 2025-07-25

**Authors:** Andrea Kun-Nemes, Dóra Farkas, Emese Szilágyi-Tolnai, Mónika Éva Fazekas, Melinda Paholcsek, László Stündl, Piroska Bíróné Molnár, Zoltán Cziáky, Judit Dobránszki, Judit Gálné Remenyik

**Affiliations:** 1 Complex Systems and Microbiome-Innovations Centre, Faculty of Agricultural and Food Sciences and Environmental Management, University of Debrecen, 4032 Debrecen, Hungary; nemes.andrea@agr.unideb.hu (A.K.-N.); tolnai.emese@agr.unideb.hu (E.S.-T.); fazekas.monika@agr.unideb.hu (M.É.F.); paholcsek.melinda@agr.unideb.hu (M.P.); molnar.piroska@agr.unideb.hu (P.B.M.); 2 Centre for Agricultural Genomics and Biotechnology, Faculty of the Agricultural and Food Science and Environmental Management, University of Debrecen, 4400 Nyíregyháza, Hungary; farkas.dora@agr.unideb.hu; 3 Institute of Food Technology, Faculty of Agricultural and Food Sciences and Environmental Management, University of Debrecen, 4032 Debrecen, Hungary; stundl@agr.unideb.hu; 4 Agricultural and Molecular Research and Service Group, Institute of Technical and Agricultural Sciences, University of Nyíregyháza, 4400 Nyíregyháza, Hungary; cziaky.zoltan@nye.hu

**Keywords:** exogenous melatonin, abiotic stress, degradation products of melatonin, potato, indole-3-acetic acid, salicylic acid

## Abstract

Plant growth regulators of natural origin are becoming increasingly important in crop production to protect plants against various abiotic stresses and often to modulate plant pathological processes. These compounds offer the potential to enhance plant health exogenously by protecting plants against oxidative stress. Melatonin has been studied previously; however, the role of exogenous melatonin in abiotic stress tolerance and the underlying mechanisms are still less understood. In this study, potato plants were grown in vitro to study the effects of exogenous melatonin and ultrasound treatment (latter as an abiotic stress). The measured parameters included morphological data and the concentrations of melatonin and its degradation products, indole-3-acetic acid and salicylic acid, at 0 h, 24 h, 1 week, and 4 weeks after treatment. In addition, the expression levels of the genes responsible for the production of enzymes involved in melatonin synthesis were traced by RT-qPCR analysis. Melatonin added to the culture medium was taken up by the in vitro plantlets, and it participated both in the plant stress reaction and stress mitigation when an abiotic stress reaction was triggered by ultrasound. Among the degradation products, we detected N-acetyl-5-methoxykynuramine, 6-hydroxymelatonin, and 5-methoxytryptamine by UHPLC-MS. Among the enzymes involved in the synthesis of melatonin and indole-3-acetic acid, the expression levels of COMT, SNAT, TSB, TAA, ASMT, TPH, AANAT, ASMT, and TSA were measured and no pattern was observed in response to the treatments.

## 1. Introduction

Environmental stress factors (salinity, drought, heat, flooding, cold) have adverse effects on plant development and crop production [1]. Abiotic stressors have been shown to be the primary cause of global crop yield loss, which can reduce average yields by more than 50% for many crops [1]. Today, one of the most important abiotic stressors is global warming, which poses a significant risk to agriculture [2]. These stressors significantly alter plant metabolism by producing reactive oxygen species (ROS) that upset ion balance and homeostasis within plants [3]. Therefore, enhancing the ability of plants to cope with these stressors is key to achieving sustainable crop production [4]. In 1995, Dubbels identified melatonin (N-acetyl-5-methoxytryptamine) as a major bioactive molecule in plants [5]. Initially, it was recognized as a potent antioxidant and has been shown to have diverse beneficial functions during different stages of plant growth and development [6], including germination [7,8], root elongation [9], photosynthesis [10] and leaf senescence [11].

### 1.1. Melatonin

Melatonin (MT) was first discovered in animals (cows) by Lerner and colleagues in 1958 and then in humans in 1959 [12,13]. Its presence in plants was only detected in 1995, but it is now accepted that MT is present in all higher plants [5,14]. It is considered a multimodal molecule as it is thought to have a number of cellular and physiological effects. As an antistressor, it increases plant tolerance to adverse environmental conditions, but it may also act as a growth regulator by regulating cell and plant growth [15]; it promotes seed germination and rooting [7,8]; optimises photosynthetic efficiency and water/CO_2_ foliar exchange; regulates the internal biological clock (flowering and ripening/ageing processes) [16,17] and regulates plant sugar metabolism [18]; and acts as an endogenous biostimulant against abiotic or biotic stressors [19,20]. Its biosynthesis is induced by abiotic stressors such as cold, drought or the presence of heavy metals, but can also be triggered by certain activated stress factors.

MT enhances plant resistance to abiotic stress in two ways, either directly by eliminating ROS molecules or indirectly by enhancing antioxidant enzyme activity, photosynthetic efficiency and metabolite abundance, and through the regulation of stress-responsive transcription factors [9,21]. Hence, the potential use of exogenous MT as a growth stimulant and stress protectant is of increasing interest [22].

The most common techniques used to measure MT are the radioimmunoassay and high-performance liquid chromatography (HPLC). Currently, HPLC coupled with mass spectrometric identification is an efficient and essential tool for the accurate determination of MT in plant samples [14,23,24,25].

### 1.2. Degradation Products of MT

**Figure 1:**
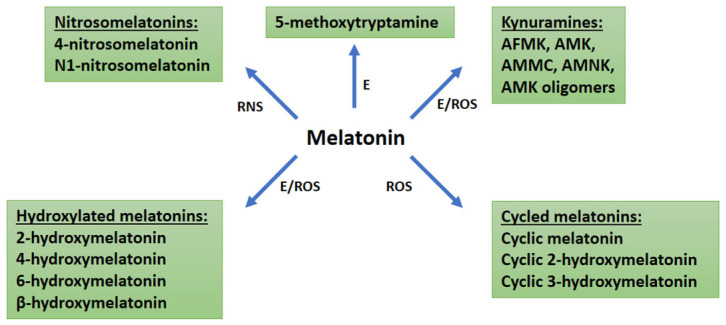
The degradation of melatonin. Abbreviations: ROS, reactive oxygen species; RNS, reactive nitrogen species; E, enzymes.

MT regulates plant stress responses both directly by inhibiting the accumulation of ROS and reactive nitrogen species (RNS) and indirectly by affecting stress response pathways. During these processes, MT is of course also transformed, producing degradation products. MT is typically degraded by enzymatic (M3H—melatonin-3-hydroxylase, M2H—melatonin-2-hydroxylase, ASDAC—N-acetylserotonin deacetylase, IDO—indoleamine-2,3-dioxygenase) or non-enzymatic (oxidants, free radicals: ROS and RNS) processes (Figure 1) [26].

### 1.3. MT and Other Phytohormones

Plant hormones, small organic molecules, are commonly involved in plant biological processes and regulate cell signalling. This category includes MT, indole-3-acetic acid (IAA) and salicylic acid (SA), which are widely distributed in the plant kingdom. All three compounds play an important role in plant physiology, especially in biotic and abiotic stress signalling and mitigation. However, they have also been described as biostimulants because they enhance photosynthetic processes and the productivity of agricultural crops [27].

IAA is the most abundant and essential auxin in plants [28]. It plays an important role in the regulation of plant growth. It regulates, for example, vascular tissue development, cell elongation and apical dominance (peak dominance) [29]. Its effects go beyond the regulation of plant growth, as it also plays roles in the regulation of bacterial physiology, adaptation to stress conditions and communication among the microbes [30].

SA is also a very important compound for plant growth and development, as it influences, among others, seed germination, seedling establishment, cell growth, respiration, stomatal closure, senescence-related gene expression, basal thermotolerance, legume nodulation and yield, as well as flowering induction and budding [31,32,33,34,35].

**Figure 2:**
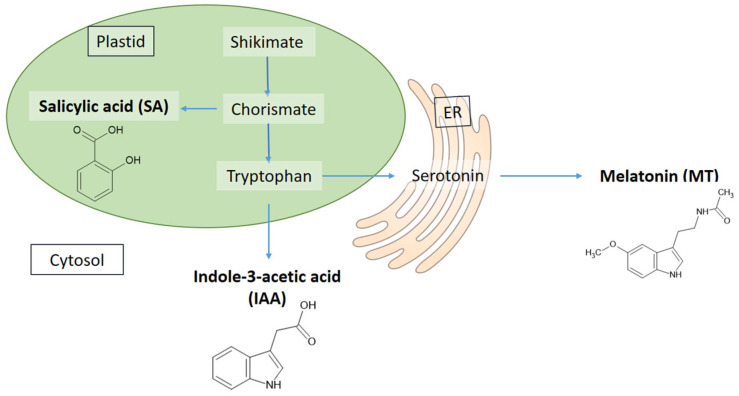
Synthesis of melatonin, indole-3-acetic acid and salicylic acid (based on [36]).

Another interesting feature is that IAA and SA share a common precursor with MT in their biosynthesis (Figure 2). Although there are many similarities in their actions, their interactions have not yet been satisfactorily studied.

The aim of our work was to investigate the effect of exogenous MT on an in vitro subculture of potato tissue cultured plantlets. We were looking for answers to the question of how it can affect plant growth, development and other phytohormones during subculture periods at various developmental times, and in addition, how MT degradation products are released, eliminated and over what time interval after applying an abiotic stressor (i.e., ultrasound treatment).

### 1.4. Quantification of MT

Various methods can be used to measure MT (bioassay, fluorescence spectrometry, gas–liquid chromatography–mass spectrometry, liquid chromatography and immunoassays). Among these techniques, the literature suggests that mass spectrometry is the most accurate and potentially the most sensitive method for MT measurement [37,38,39]. However, as this method of measurement requires expensive equipment and highly trained personnel and it is not suitable for the measurement of large sample numbers, a series of commercial enzyme-linked immunosorbent assay (ELISA) kits are now available.

As there is no uniform isolation and measurement protocol in scientific publications, differences on the order of magnitude for the same plant can be observed [40]. However, in order to compare the results of different working groups, it is important to develop and apply a single measurement method.

### 1.5. Enzymes Involved in the Synthesis of MT and IAA

**Figure 3:**
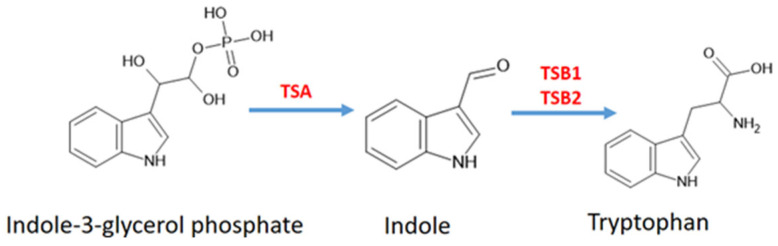
Synthesis pathway of tryptophan. Abbreviations: TSA, tryptophan synthase A; TSB, tryptophan synthase B.

Tryptophan (Trp) is the common precursor of both MT and IAA. Trp is formed from indole-3-glycerol phosphate via indole. This process is catalysed by TSA (tryptophan synthase A), TSB1 and TSB2 (tryptophan synthase B1, B2) (Figure 3).

**Figure 4:**
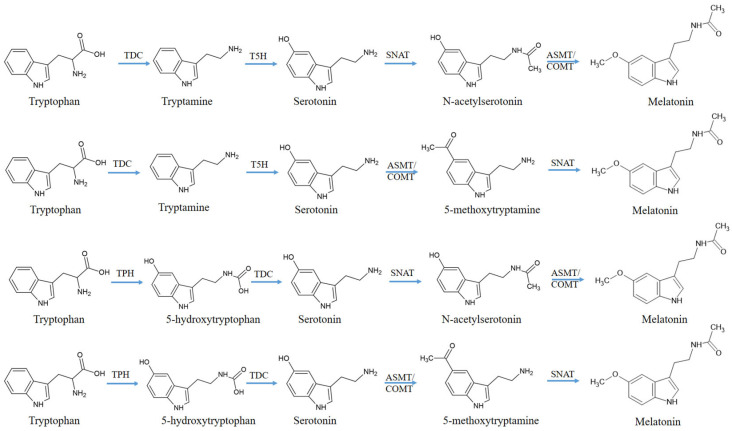
Synthesis pathways of melatonin from tryptophan metabolism [43]. Abbreviations: TDC, tryptophan decarboxylase; T5H, tryptamine 5-hydroxylase; TPH, tryptophan hydroxylase; AANAT, aril-alkil-amin-N-acetyltransferase; SNAT, serotonin N-acetyltransferase; ASMT, acetylserotonin O-methyltransferase; COMT, caffeic acid O-methyltransferase.

MT biosynthesis begins with Trp and consists of four enzymatic steps, but at least six enzymes are known to be involved, such as tryptophan decarboxylase (TDC), tryptophan hydroxylase (TPH), tryptamine 5-hydroxylase (T5H), serotonin N-acetyltransferase (SNAT), N-acetylserotonin methyltransferase (ASMT), and caffeic acid O-methyltransferase (COMT). In addition, there exist multiple (at least four) biosynthetic pathways (Figure 4). All four different routes for MT biosynthesis necessarily involve serotonin as an intermediate, suggesting that serotonin is an essential intermediate for MT synthesis in plants [41]. AANAT (arylalkylamine N-acetyltransferase) is also named serotonin N-acetyltransferase (SNAT); therefore, to differentiate them from animal AANAT genes, plant AANAT genes are frequently termed SNAT genes [42].

**Figure 5:**
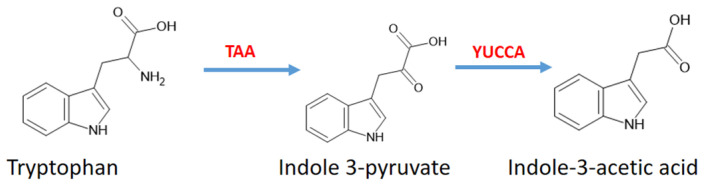
Synthesis pathway of IAA in tryptophan metabolism [44]. Abbreviations: TAA, tryptophan aminotransferase.

IAA is also biosynthesized from Trp through the indole-3-pyruvic acid pathway, which involves tryptophan aminotransferase TAA and YUCCA enzymes (Figure 5).

## 2. Materials and Methods

At least 3 replicates were used for each preparation and measurement.

### 2.1. MT and Ultrasound Treatments of In Vitro Plant Material

Single-node segments of 4-week-old in vitro plantlets of potato (*Solanum tuberosum* L. cv. Desirée) were used in the experiments. The in vitro culture conditions for culturing the plantlets were the same as those used in previous experiments, i.e., 22 ± 2 °C under a 16 h photoperiod at a photosynthetic photon flux density of 63.5 μmol m^−2^ s^−1^ [45].

Exogenous MT, ultrasound treatment (US) or a combination of these (MTUS) were used to treat the plant material. MT was added to the Murashige and Skoog (MS) [43] culture medium at 50 µM before autoclaving for the MT and MTUS treatments. When ultrasonication was applied (US and MTUS treatments), the excised single-node segments were ultrasonicated (35 kHz, 70 W) for 20 min with an Elmasonic X-tra 30 H ultrasonicator, at 20 °C) as described earlier [44,45,46] and before culturing them on MS medium. Then, the plant materials were cultured in vitro for a subculture (for 4 weeks) under the same conditions as the starting plant material.

### 2.2. Sampling Times and Measurements of Morphological Parameters and Chlorophyll Content

Plant samples were taken at 0 h, 24 h, 1 week and 4 weeks after the subculture initiation and they were stored at −80 °C until biochemical analysis and RNA-Seq. At least 90 explants (at 0, 24 and 48 h) or plantlets (at 1 week and 4 weeks) were used from 3 different jars for sampling.

Lengths and fresh weights of shoots and roots, and the number of nodes per plantlet were measured 1 week after the start of the experiments and at the end of the in vitro subculture (at 4 weeks). Furthermore, the number of shoots per explant and the branching of the shoots were also observed. In the latter two cases, only shoots that reached the average shoot length in each treatment were considered. Chlorophyll a (chl a), chlorophyll b (chl b) and chl a + chl b content in shoots of 4-week-old plantlets were measured spectrophotometrically and then the ratio of chl a/chl b was calculated as described earlier [47,48]. Plant samples were taken from 5 jars, each containing 30 explants or plantlets, for each measurement, which means 5 × 30 = 150 replicates for measurements from explants or plantlets, and 5 replicates if parameters were measured per jar for each treatment and sampling time. Chlorophyll contents were measured from 6 jars per treatment.

### 2.3. ELISA

MT levels in potato leaf samples were measured using an ELISA kit (#ab213978, Abcam, Cambridge, MA, USA) according to the manufacturer’s instructions.

### 2.4. Sample Preparation for UHPLC-MS

The plant sample (300 mg) was triturated in liquid nitrogen and extracted with 5 mL of methanol at room temperature for 40 min in an ultrasonic bath. Following centrifugation (4 °C, 4500 rpm, Eppendorf Centrifuge 5810R, Eppendorf, Schönenbuch/Basel, Switzerland), the supernatant was evaporated (Heidolph Hei-VAP Value rotary evaporator, Schwabach, Germany). The extracted compounds were redissolved in 1 mL of 5% methanol and purified on a Supelclean ENVI-18 SPE column (Supelco, Washington, DC, USA). The column was regenerated with methanol and water and washed with 5% methanol after adding the sample. Elution was performed with 80% methanol. The sample extracted from the column was evaporated and stored in a freezer (−20 °C) until measurement.

### 2.5. UHPLC-MS Analysis

The UHPLC system (Dionex Ultimate 3000RS, Thermo Scientific, Waltham, MA, USA) was coupled to a Thermo Q Exactive Orbitrap mass spectrometer (Thermo Fisher Scientific Inc., Waltham, MA, USA) equipped with an electrospray ionization source (ESI). The UHPLC separation was achieved on a Phenomenex Kinetex XB-C18 (100 mm × 2.1 mm, i.d. 2.6 μm). The sampler and oven temperature were maintained at 25 °C and the flow rate was 0.2 mL min^−1^. The mobile phase consisted of methanol (A) and water (B) (both acidified with 0.1% formic acid). The gradient program was as follows: 0–3 min, 95% B; 3–11 min, 0% B; 11–20 min, 95% B. The injection volume was 2 μL. The mass spectrometer was equipped with an ESI source, and the samples were measured in positive ionization mode. The resolution was set to 35,000. The mass range scanned was 100–1500 *m*/*z*. The maximum injection time was 100 ms. The resolution was set to 17,500 in the cases of MS2 scans. The collision energy was 20; 30; 40 NCE. Xcalibur 4.0 (Thermo Fisher Scientific Inc., Waltham, MA, USA) software was used to collect and analyse data.

### 2.6. Salicylic Acid

The biosensor (*Acinetobacter* sp. ADPWH_lux) and the method developed by Huang et al. were used for the measurement of SA with modifications by DeFraia et al. [49,50,51].

### 2.7. RNA Isolation

Total RNA isolation from potato samples was performed according to the Invitrogen user guide with an EasySC Universal RNA Purification Kit (cat. number: R137-2) from ABP Biosciences (Rockville, MD, USA). The exact procedure for using the kit is available on the distributor’s website. The RNA was stored at −80 °C until used in further experiments.

The quality and concentration of RNA samples were determined using a Nanodrop 2000 spectrophotometer (Thermo Scientific, MA, USA).

### 2.8. RT-qPCR Reaction

Total RNA from each potato plant was quantified using a Nanodrop 2000 spectrophotometer. RNA was reverse-transcribed into cDNA using dark blue LunaScript RT SuperMix (New England BioLabs, Ipswich, MA, USA) for qPCR reactions. A FrameStar 96 Well Semi-Skirted PCR Plate was used for the operation.

qPCR was performed on a Roche LightCycler^®^ 96 Instrument (Roche, Basel, Switzerland). The amplification was performed using the Luna Universal Probe qPCR Master Mix assay (New England Biolabs). The *β-tubulin* housekeeping gene was used as an internal control. Genes of interest were selected based on their relevance to this study and primers were designed and obtained from Metabion International AG (Planegg, Germany) (Table 1). Since no breed-identical genes were available and since they were not cloned or sequenced in their genotypes, they can only be considered as putative genes. Both SYBR^®^ Green and TaqMan^®^ primers were used in the study. The genes were not cloned in this experiment. The amplification protocol consisted of an initial denaturation step at 95 °C for 60 s, followed by 40 cycles at 95 °C for 15 sec and optimal annealing temperature (60 °C) for 30 s.

The obtained CT values were converted into fold change values using the ∆CT method.

### 2.9. Statistical Analyses

One-way ANOVA followed by Tukey’s test at *p* < 0.05 using SPSS for Windows software (SPSS^®^, version 21.0) were used for analysing the morphological and chlorophyll content data of 1- and 4-week-old plantlets. Data are expressed as the mean ± standard error. To evaluate the difference between groups, data were analysed by multivariate (ANOVA) using GraphPad 8.0. Heatmaps were generated in R statistical software (ver. 4.2.2) using the heatmap package.

## 3. Results

### 3.1. Effects of the Exogenous MT and After-Effects of Ultrasonication on the Growth Parameters and Chlorophyll Content of In Vitro Potato Plantlets

All measured morphological parameters of 1-week-old plantlets were significantly affected by the applied treatments (Table 2). Exogenously applied MT significantly decreased all morphological parameters. While the lengths of shoots and roots decreased by 46% and 49%, respectively, and the decrease in the shoot fresh weight was 38%, a particularly significant decrease of 73% could be detected in the fresh weight of roots. The decrease in the number of nodes per plantlet was only 19%. Ultrasonication tended to improve the fresh weights of shoots and roots (by 46% and 20%, respectively); however, the differences were not proven to be statistically significant. When ultrasonicated explants were cultured on MT-containing medium (MTUS treatment), the shoot and root lengths and the number of nodes per plantlet of the 1-week-old plantlets significantly increased (by 39%, 69%, and 47%, respectively) compared to plantlets grown on MT-containing medium but without prior ultrasonication of the explants. The shoot and root lengths of 1-week-old plantlets from combined treatment (MTUS), however, could not reach the values achieved in control or only ultrasonicated plantlets. The highest number of nodes per plantlets was achieved with the combined treatment (MTUS) compared to any of the other treatments or control. Considering the number of shoots that reached the average shoot length in each treatment, the average shoot number per explant significantly decreased in response to the exogenous MT supplement in the medium (Table 2). Branching of shoots could not be detected in either the control or the combined treatment, but it was observed in the US and MT treatments, by 11% and 31%, respectively.

Significant differences in all morphological parameters and the chlorophyll content of the 4-week-old shoots were detected in response to the different treatments at the end of the subculture (Table 3). US treatment applied alone or in combination (MTUS) significantly increased the shoot weight and the length of the roots but decreased the shoot length. MT supplementation in the medium caused a significant decrease in the weight of the roots and in the shoot length, and in combination with US (MTUS), a further decrease was detected in the shoot length. However, the sole application of MT led to a significant increase in the number of nodes per shoot and in the number of shoots per explant. When it was combined with US (MTUS), its shoot number increasing effect was eliminated while it decreased the number of nodes per shoot below the value of the control.

MT treatment caused a drastic, about three-fold increase in the chl a, chl b and chl a + chl b contents. In the combined treatment (MTUS), the effect of MT in increasing the chlorophyll contents was slightly smaller and approximately 2.5-fold increases were detected due to the effect of the US, which tended to decrease the measured chlorophyll content of the shoots. The ratio of chl a/chl b did not change in the various treatments.

### 3.2. Melatonin Concentration by ELISA and UHPLC-MS

**Figure 6:**
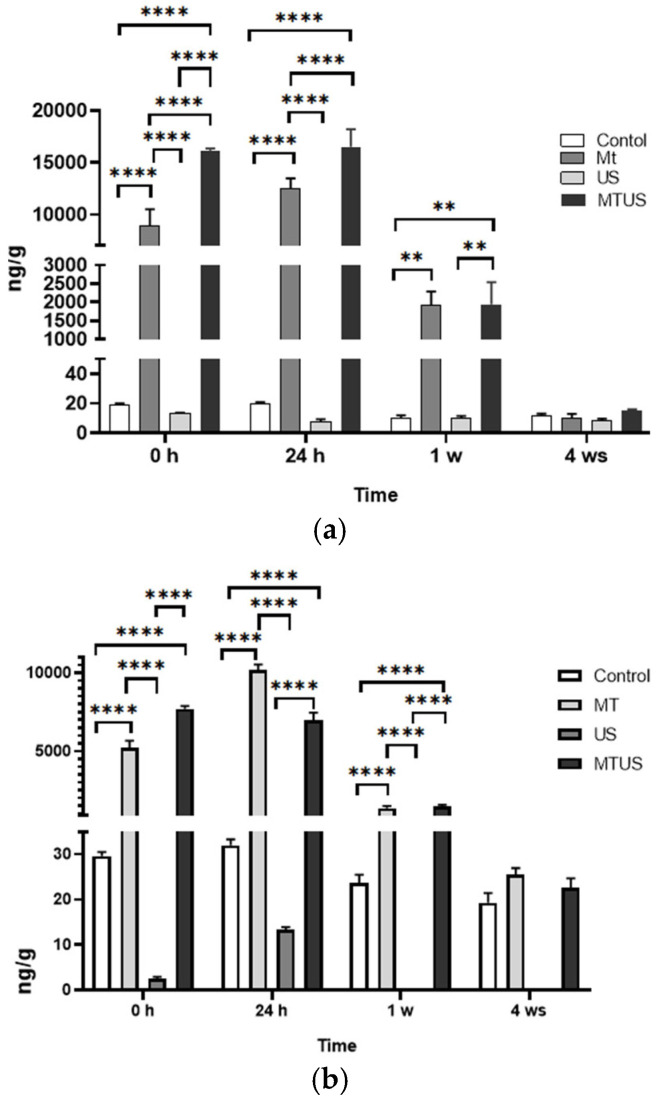
Melatonin concentration by ELISA (**a**) and UHPLC-MS (**b**). Abbreviations: melatonin-containing medium (MT), ultrasound treatment (US) and combined treatment (MTUS). Statistics: no difference *p* > 0.05; ** *p* ≤ 0.01; **** *p* ≤ 0.0001.

MT concentrations were measured by ELISA and UHPLC-MS. Based on the control sample, we can see the normal course of the MT concentration. In the case of ELISA (Figure 6a), no major deviations were observed during the experimental period. The average MT concentration over the whole period was 14.84 ng/g. When the plantlets were treated with US, the MT concentration did not change significantly, thus the treatment had no effect on MT levels. The mean concentration value during the experiment was 9.83 ng/g. The MT concentration of the 0 h and 24 h samples treated with MT was 10,732 ng/g. As expected, a significant part of the MT in the medium was absorbed by the plantlets, so that in these cases the concentration of MT was already significantly increased at 0 h. An increased value was even observed in the 24 h samples. After 1 week, the concentration of MT started to decrease. The extent of the reduction in the 24 h value was 15.42% for MT and 11.78% for MTUS. At the end of the experimental period, MT levels returned to the concentration typical of the control plant, regardless of whether MT or US was used.

**Figure 7:**
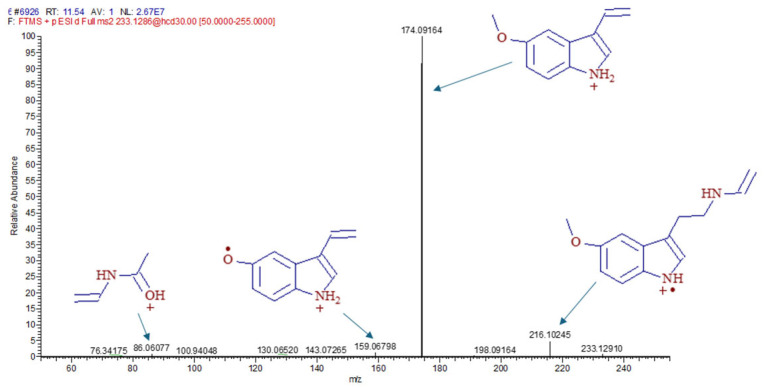
The fragmentation spectrum of melatonin.

In case of UHPLC-MS (Figure 6b) by the 4th week, for the mean value of MT in the control samples (26.13 ng/g) and in the US treatment (3.93 ng/g), this measurement method did not show any significant difference. In the MT-treated samples, the increase in this case also appeared at 0 h and was also observed at 24 h. After 1 week, MT levels had already decreased significantly in the MT and MTUS samples (by 13.37% and 21.27%, respectively) and reached a concentration typical of control plant levels by the end of the experiment. The fragmentation spectrum of MT is shown in Figure 7.

Our results could only be compared with our own previous paper, as we could not find any other literature on the use of exogenous MT in culture media [46].

### 3.3. Melatonin Degradation Products

In order to evaluate the quantitative changes in degradation products more accurately, these values were plotted as a function of time, not only per degradation product but also per treatment. N-acetyl-N-formyl-5-methoxykynuramine (AFMK) could not be detected in either sample, suggesting that AFMK produced from MT was immediately further degraded to AMK.

**Figure 8:**
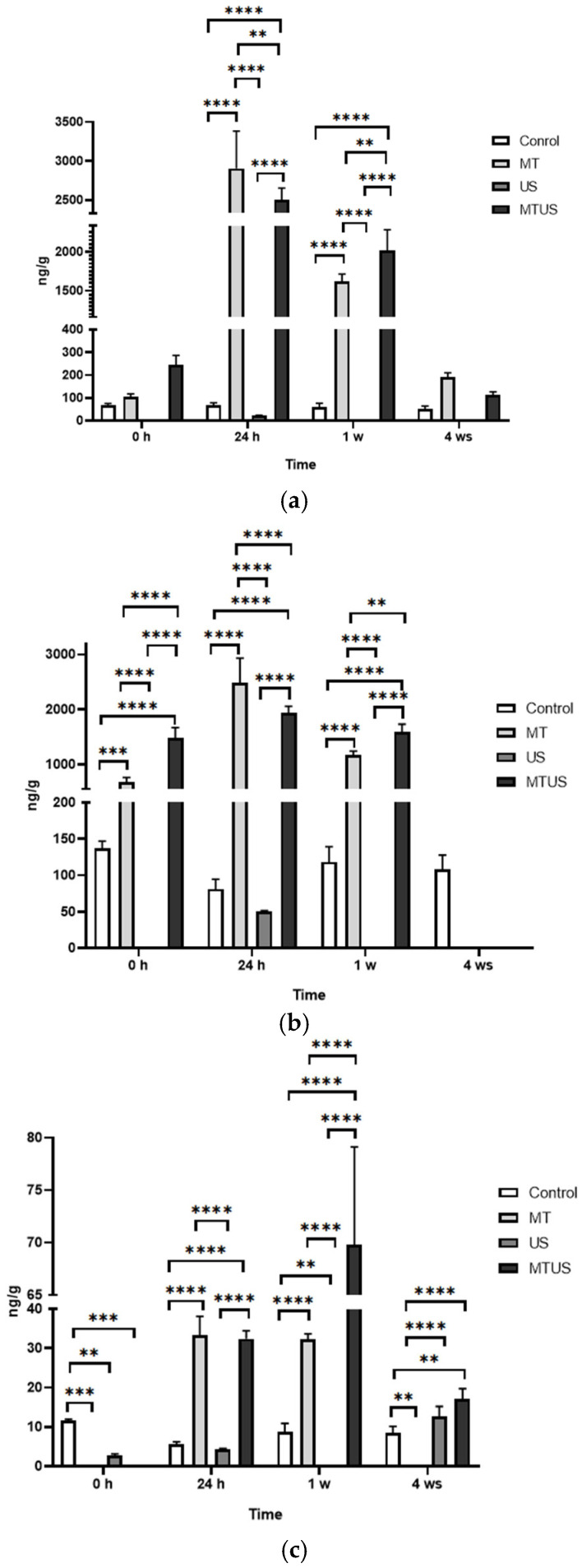
The degradation products of melatonin. AMK (**a**), 6HM (**b**), 5MTA (**c**). Abbreviations: melatonin-containing medium (MT), ultrasound treatment (US) and combined treatment (MTUS), N-acetyl-5-methoxykynuramine (AMK), 6-hydroxymelatonin (6HM), 5-methoxytryptamine (5MTA). Statistics: no difference *p* > 0.05; ** *p* ≤ 0.01; *** *p* ≤ 0.001; **** *p* ≤ 0.0001.

In the time point comparison (Figure 8), we see that while the plantlets absorbed MT already at 0 h, its degradation was mostly observed only from 24 h. Of the three degradation products detected, two forms, AMK (Figure 8a) and 6HM (Figure 8b), were present in higher amounts (3414 and 2990 ng/g in the 24 h MT sample, respectively). In comparison, 5MTA (Figure 8c) was present in negligible amounts (mean value 69.8 ng/g in the 1 week MTUS sample); thus, it can be concluded that the two typical degradation products were AMK and 6HM.

The amount of AMK did not change significantly in the 0 h sample but it increased significantly in the MT and MTUS samples in the next two stages before decreasing back to baseline by week 4. Compared to the control 0 h mean (69.06 ng/g), the maximum value was 49.44-fold for MT and 37.88-fold for MTUS. In the case of US, no AMK was detected at any time point except for the 24 h sample. At this time point, the mean of the measured values was 23.50 ng/g.

**Figure 9:**
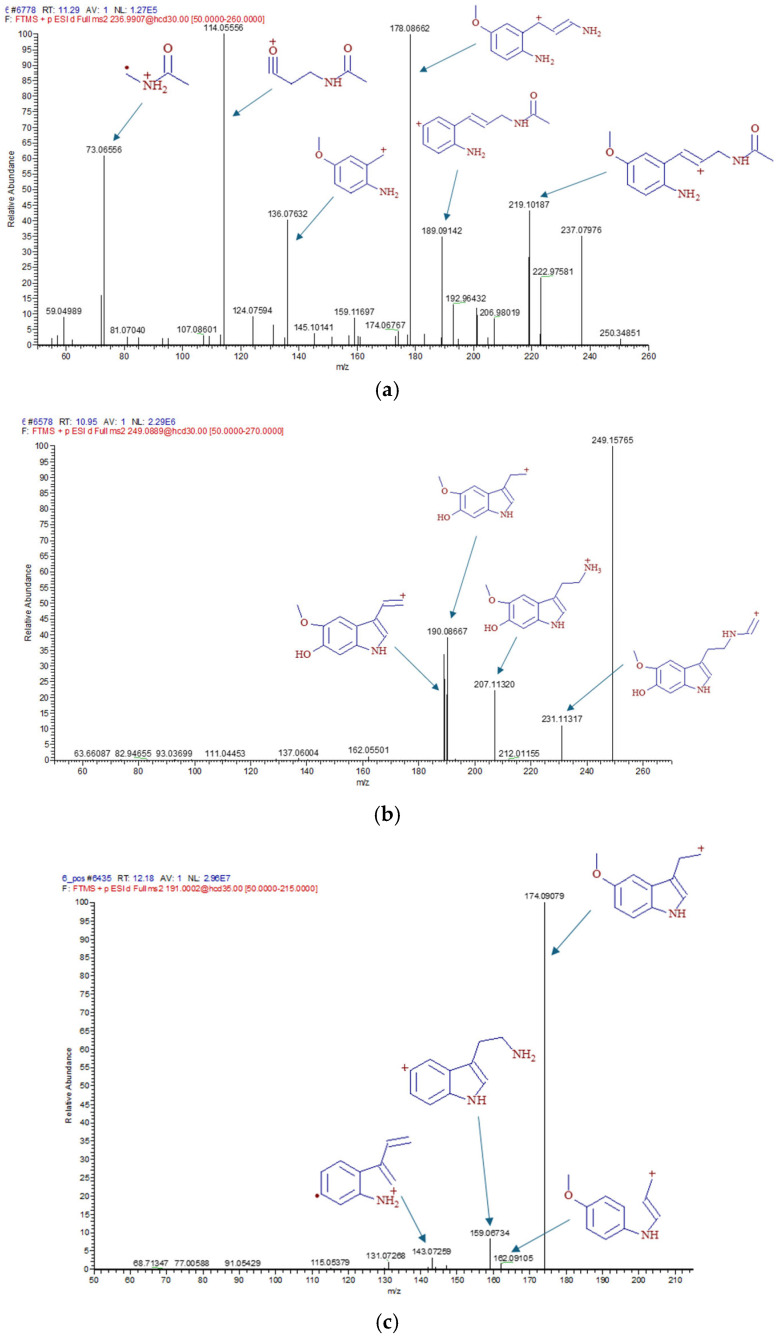
Fragmentation spectrum of melatonin and melatonin degradation products. AMK (**a**), 6HM (**b**), 5MTA (**c**). Abbreviations: N-acetyl-5-methoxykynuramine (AMK), 6-hydroxymelatonin (6HM), 5-methoxytryptamine (5MTA).

The presence of 6HM was surprising, as it has been reported in several articles that this form is characteristic of animals. 6HM has been found in liver and kidney tissues, and has also been detected in multiple biofluids, such as urine and blood [26,52,53]. Nevertheless, since the fragmentation spectra (Figure 9) obtained by UHPLC-MS were consistent with the standard and the sample, we do not question this result.

6HM appeared as early as 0 h and, like AMK, reached its maximum at 24 h and then disappeared at 4 weeks, with a steady decline. Compared to the 0 h mean of the control (137.67 ng/g), the maximum value was 21.72-fold for MT, whereas for MTUS, the difference was 14.99-fold. In the case of the US treatment, 6HM was present only in the 24 h sample and the mean value of the measurements was 50.04 ng/g.

A significant decrease in 5MTA was observed in the 0 h samples compared to the control in all treatments. At 24 h, 5MTA levels in the US sample returned to that of the control (US 4.25 ng/g), while MT treated samples showed a significant increase (MT: 33.35 ng/g, MTUS: 32.45 ng/g). This value remained the same for MT in the 1 week sample (32.38 ng/g), while MTUS continued to increase significantly (69.8 ng/g). By week 4, the levels were significantly reduced, no longer detectable in the MT sample, but still significantly higher in the US and MTUS samples than in the control sample, even after the reduction.

**Figure 10:**
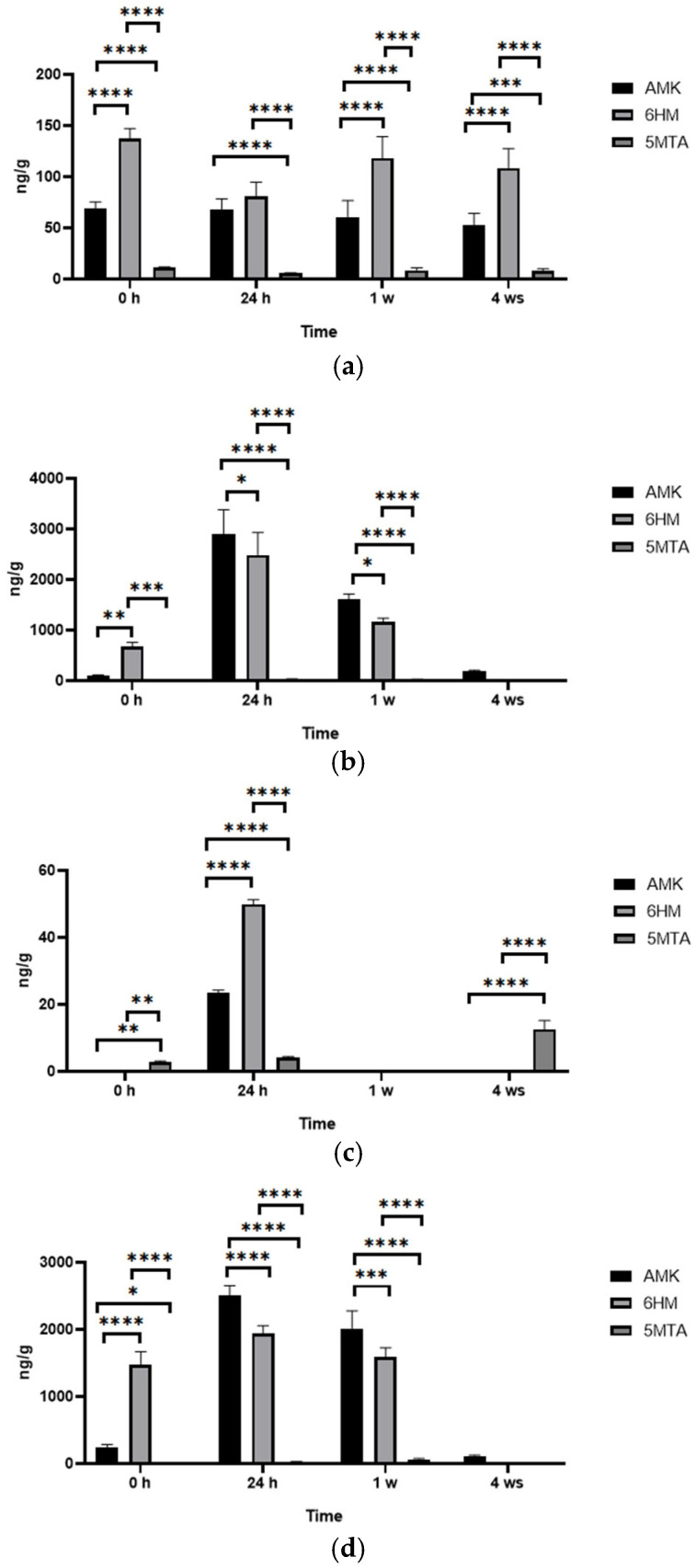
The amount of melatonin degradation products. Control (**a**), MT (**b**), US (**c**), MTUS (**d**). Abbreviations: melatonin-containing medium (MT), ultrasound treatment (US) and combined treatment (MTUS). N-acetyl-5-methoxykynuramine (AMK), 6-hydroxymelatonin (6HM), 5-methoxytryptamine (5MTA). Statistics: no difference *p* > 0.05; * *p* ≤ 0.05; ** *p* ≤ 0.01; *** *p* ≤ 0.001; **** *p* ≤ 0.0001.

If we look at the data by treatment, we can see for the control sample how the values for each of the degradation products change during normal plant growth under in vitro conditions. The continuous variation of these values indicates that the development and growth of plant parts is a high biotic stress. All three decomposition products were present at all time points studied, but 6HM was the most abundant decomposition product (Figure 10a). It can be seen that in MT samples (MT (Figure 10b) and MTUS (Figure 10c)), the proportions of 6HM and AMK changed, with AMK being the most abundant decomposition product. The least amount of degradation product was characteristic of this US (Figure 10d) treatment. Interestingly, in this case at 0 h and 4 w time points, 5MTA was the characteristic degradation product, and in the 1 week sample, none of the degradation products were detectable.

The effect of exogenous MT followed the same pattern in both treatments. In the 0 h, only AMK and 6HM were present, and 6HM had a higher concentration. By 24 h, the amounts of AMK and 6HM were significantly increased (AMK: MT 2905 ng/g, MTUS 2503 ng/g; 6HM: MT 2492 ng/g, MTUS 1937 ng/g) and proportionally, AMK was already higher. Their quantity showed a decreasing trend by week 1 and the predominance of AMK was still observed (AMK: MT 1619 ng/g, MTUS 2018 ng/g; 6HM: MT 1172 ng/g, MTUS 1592 ng/g). By week 4, the decline continued to the point that virtually only AMK was detectable, but even that was at low concentrations (MT 190.33 ng/g, MTUS 113.85 ng/g). The correctness of the identified components is confirmed by the fragmentation spectra (Figure 10).

**Figure 11:**
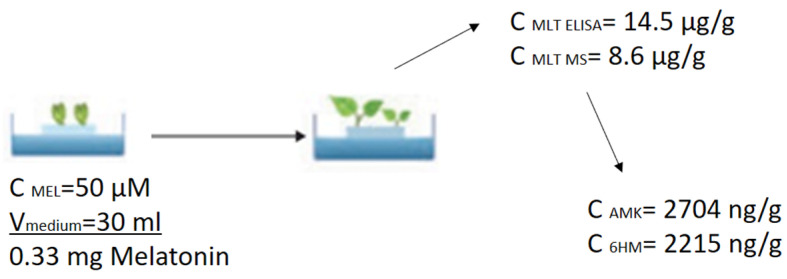
Quantitative relationships between melatonin and melatonin breakdown products. The concentration values are the cumulative average values of the 1 week MT and MTUS samples. Abbreviations: melatonin-containing medium (MT), combined treatment (MTUS).

In terms of quantitative relationships, it can be said that the plants absorbed 14.5 µg/g and 8.6 µg/g according to the measurements by ELISA and UHPLC-MS, respectively, from the medium containing 0.33 mg MT, which was mainly degraded to AMK and 6HM during the first week (Figure 11).

### 3.4. Indole-3-Acetic Acid (IAA)

Based on the literature, MT and IAA can both regulate each other’s levels; however, the mechanism of MT regulation in plants from the perspective of IAA is not yet known. Both hormones are known to interact extensively with other hormone signalling pathways by regulating their biosynthesis or signalling [54].

**Figure 12:**
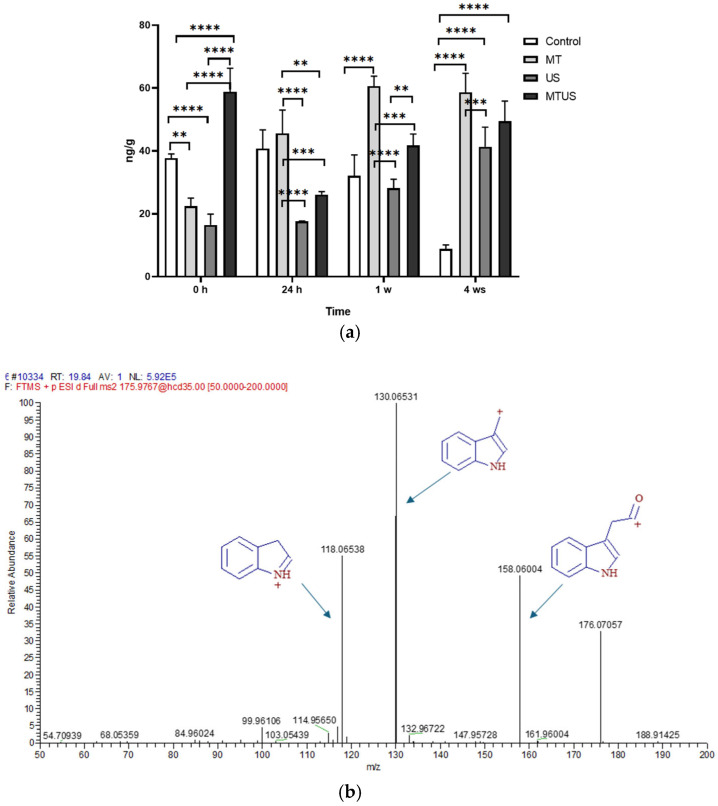
IAA concentration by UHPLC-MS (**a**) and its fragmentation spectrum (**b**). Abbreviations: melatonin-containing medium (MT), ultrasound treatment (US) and combined treatment (MTUS). Statistics: no difference *p* > 0.05; ** *p* ≤ 0.01; *** *p* ≤ 0.001; **** *p* ≤ 0.0001.

It can be seen that normal plant growth requires a higher amount of IAA (39.23 ng/g), mainly in the first 24 h, after which there is a continuous decrease (Figure 12a). While it is still 32.05 ng/g in week 1, only 8.82 ng/g is required in week 4. That means that the initial amount was reduced by a quarter by week 4. When only MT or only US were used at 0 h, IAA responded by reducing its concentration, but when both treatments were used together (MTUS), high IAA production was observed. After that, a continuous increase was seen in the MT and US samples. In the case of MTUS samples, the initial high value (58.84 ng/g) dropped to 26.09 ng/g by 24 h, but then there was an increase again in the 1st and 4th weeks. The fragmentation spectrum of IAA is shown in Figure 12b.

Based on our measurements, MT increased significantly starting almost at 0 h and then was continuously depleted from the plant tissues. In parallel, the amount of IAA continuously increased. As we saw earlier (Figure 6), MT increased significantly starting almost at 0 h and then was continuously depleted from plant tissues. We observed that in parallel, the amount of IAA continuously increased.

### 3.5. Salicylic Acid

**Figure 13:**
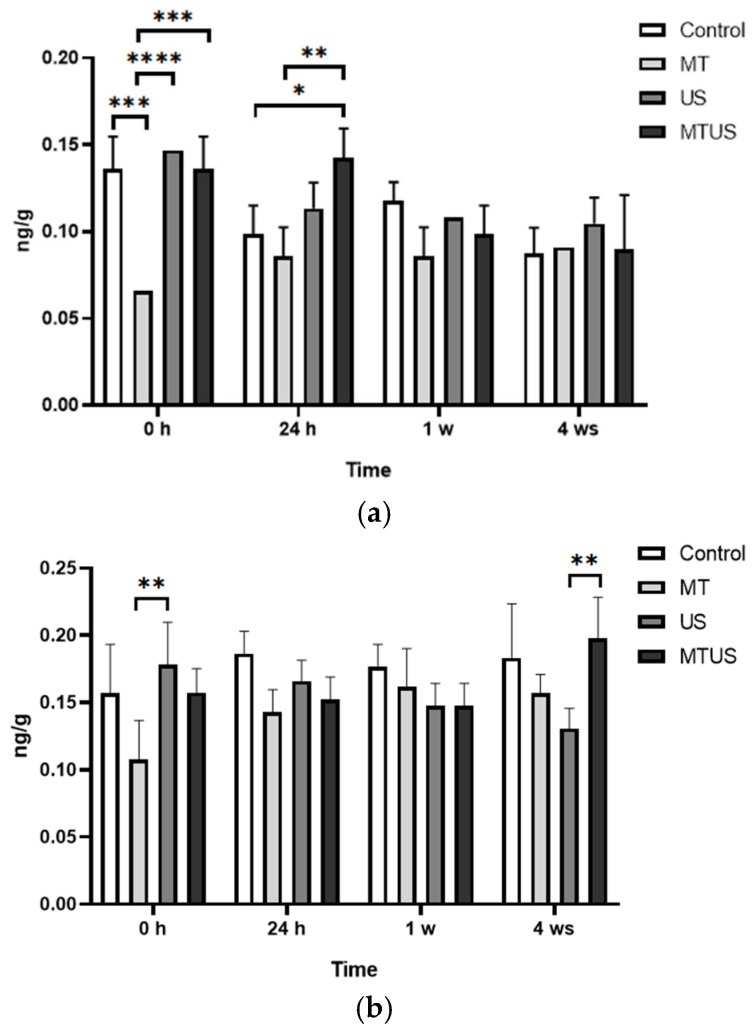
Salicylic acid concentration by biosensor method. Free salicylic acid (**a**), free and bound salicylic acid together (**b**). Abbreviations: melatonin-containing medium (MT), ultrasound treatment (US) and combined treatment (MTUS). Statistics: no difference *p* > 0.05; * *p* ≤ 0.05; ** *p* ≤ 0.01; *** *p* ≤ 0.001; **** *p* ≤ 0.0001.

Free SA (Figure 13a) decreased significantly in the MT samples at 0 h, but there was no change in the US and MTUS samples compared to the control. By 24 h, the value measured for the control decreased, MT increased in parallel, and US caused a decrease to such an extent that there was no longer a significant difference among the three treatments. Only the MTUS sample showed an outlier. By the first week, the values normalized, and this did not change even in the 4th week.

If we examine the amount of free and bound salicylic acid together (Figure 13b), the results are much more balanced. We found only two significant differences. One can be observed at 0 h, when the value measured for the US sample was significantly higher than for the MT sample. The other was seen at 4 weeks, when the value of MTUS was significantly higher than US. The fragmentation spectrum of IAA is shown in Figure 13b.

### 3.6. Relative Fold Change Analysis Compared to Control

The enzymes required for Trp synthesis are TSA, TSB1 and TSB2. In the next stage of the synthesis, as shown in Figure 4, MT is formed from Trp via serotonin. For this, the conversion can occur via four different pathways and the required enzymes are TDC, TPH, T5H, SNAT, ASMT and COMT. Among these, we were unable to detect the genes for the TDC and T5H enzymes. Arylalkylamine N-acetyltransferase or serotonin N-acetyltransferase (SNAT) is also known as aralkylamine N-acetyltransferase (AANAT). We purchased two sets of qPCR primers under two different names, and we kept the original name.

#### 3.6.1. TSA Gene

**Figure 14:**
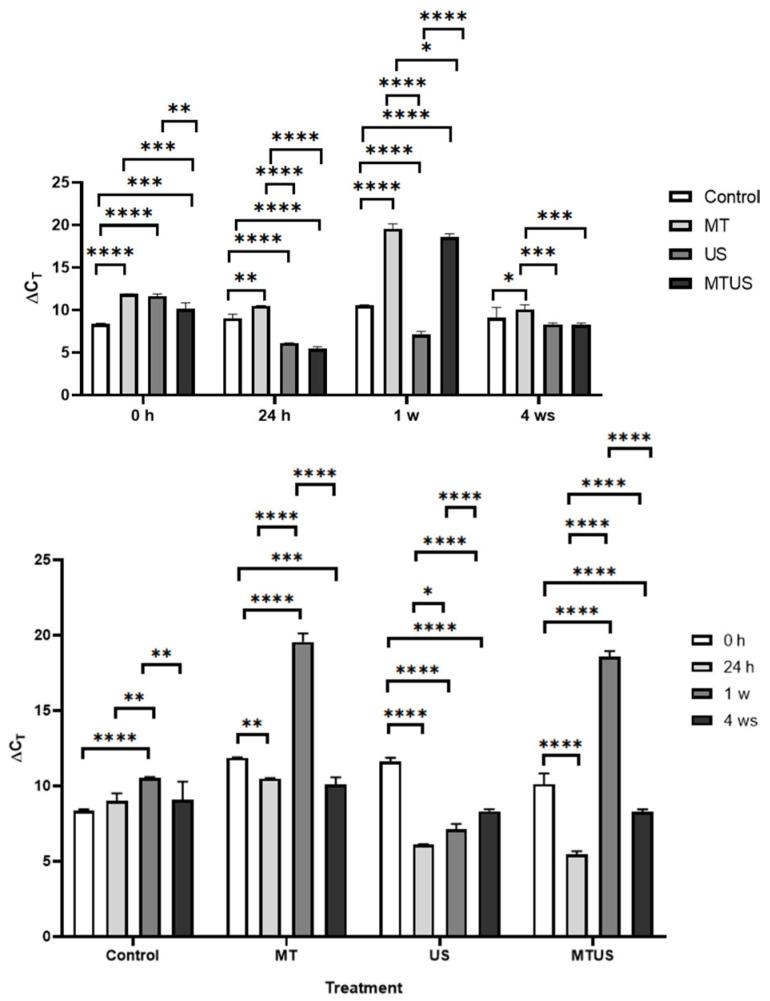
TSA expression levels. Abbreviations: melatonin-containing medium (MT), ultrasound treatment (US) and combined treatment (MTUS). Statistics: no difference *p* > 0.05; * *p* ≤ 0.05; ** *p* ≤ 0.01; *** *p* ≤ 0.001; **** *p* ≤ 0.0001.

In the case of *TSA* (Figure 14), at 0 h, a significantly increased level was measured compared to the control in all three treatments. However, by 24 h, only MT showed an increased expression level, while in response to US and MTUS, its expression level became significantly lower. The most interesting result can be observed in the 1 week samples. In this case, the MT and MTUS samples showed an outstanding value, which was 1.86- and 1.76-times higher compared to the control value, respectively. Due to the presence of a large amount of MT, the plant is not required to produce MT during this period, and therefore, a more likely explanation seems to be that this result was related to the quantitative increase in IAA. However, it can also be stated that the values began to reach the concentration typical of control plants by 4 weeks.

#### 3.6.2. TSB1 and TSB2 Genes

**Figure 15:**
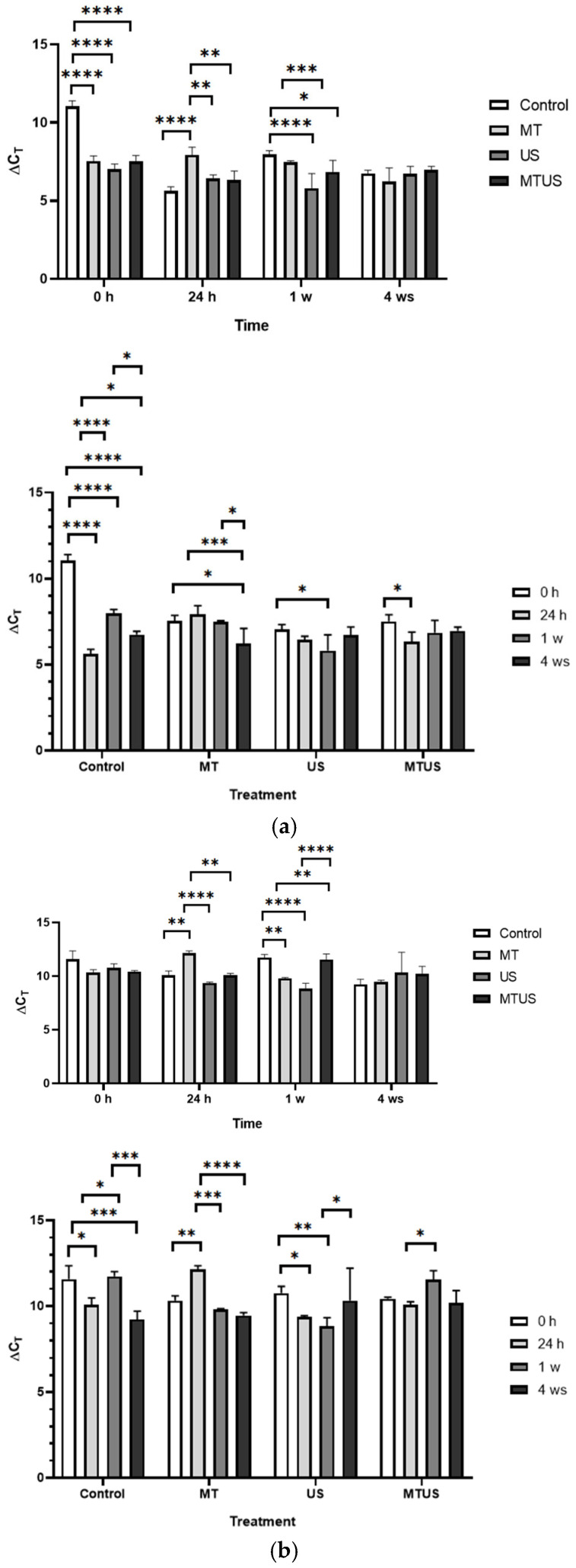
The expression levels of TSB1 (**a**) and TSB2 (**b**). Abbreviations: melatonin-containing medium (MT), ultrasound treatment (US) and combined treatment (MTUS). Statistics: no difference *p* > 0.05; * *p* ≤ 0.05; ** *p* ≤ 0.01; *** *p* ≤ 0.001; **** *p* ≤ 0.0001.

The time course of both enzymes was very similar. In the case of *TSB1* (Figure 15a) and *TSB2* (Figure 15b), we could no longer detect an outlier value, similar to *TSA*. In this case, values close to or below the control were more likely. The number of outstanding values during the treatments was minimal, such as the 24 h MT (7.93) in the case of *TSB1*, and the 24 h MT (12.14) and the 1 week MTUS (11.54) in the case of *TSB2*. By the end of the experimental period, the levels of the gene expression in the control and treated samples also became uniform (*TSB1*: 6.66; *TSB2*: 9.80).

Since Trp is a common precursor of another biologically active molecule in addition to MT and IAA [55], we did not expect a close correlation with elevated MT levels at this stage of the synthetic pathway.

#### 3.6.3. TPH Gene

**Figure 16:**
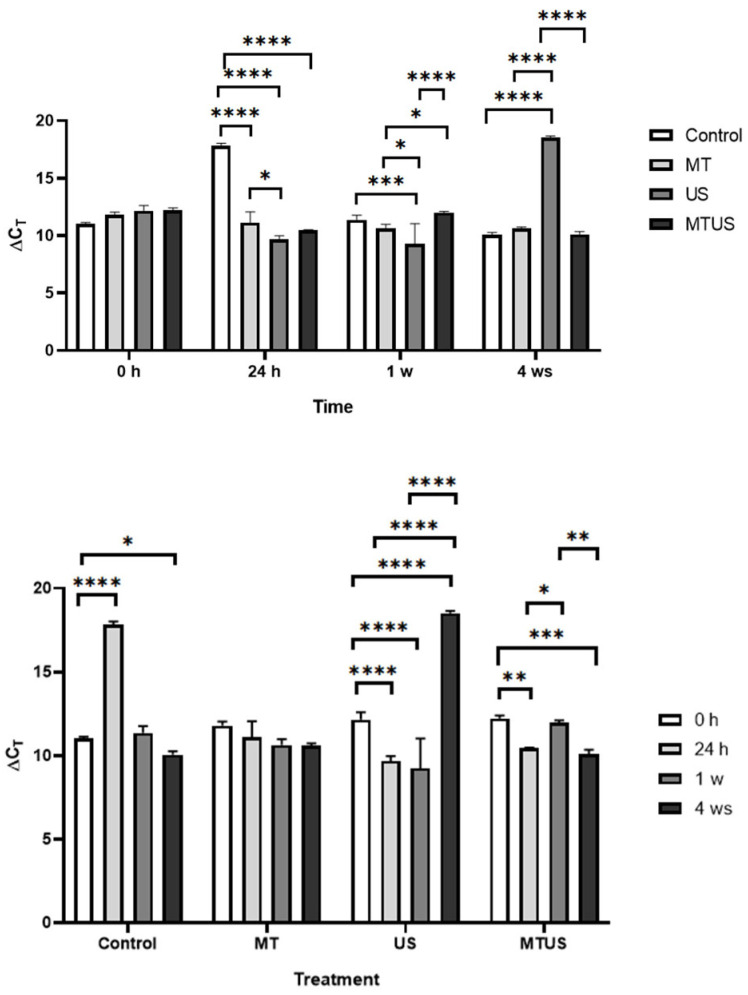
THP expression levels. Abbreviations: melatonin-containing medium (MT), ultrasound treatment (US) and combined treatment (MTUS). Statistics: no difference *p* > 0.05; * *p* ≤ 0.05; ** *p* ≤ 0.01; *** *p* ≤ 0.001; **** *p* ≤ 0.0001.

We did not observe any significant differences in the gene expression of this enzyme at the initial time point (Figure 16). At 24 h, we found a significant increase in the control, so compared to this, the plants showed reduced expression after the treatments. The results of the US samples were interesting, since in the first week it was significantly lower than the others (9.25), but by 4 weeks it reached a very high value (18.53). The other values did not differ significantly from each other.

#### 3.6.4. AANAT and SNAT Genes

We obtained different results for gene expression of these two enzymes in terms of time course and quantity. In the case of AANAT, the highest value was 13.04 (1 week, MT samples), while in the case of *SNAT*, 5.64 was measured at the same time.

**Figure 17:**
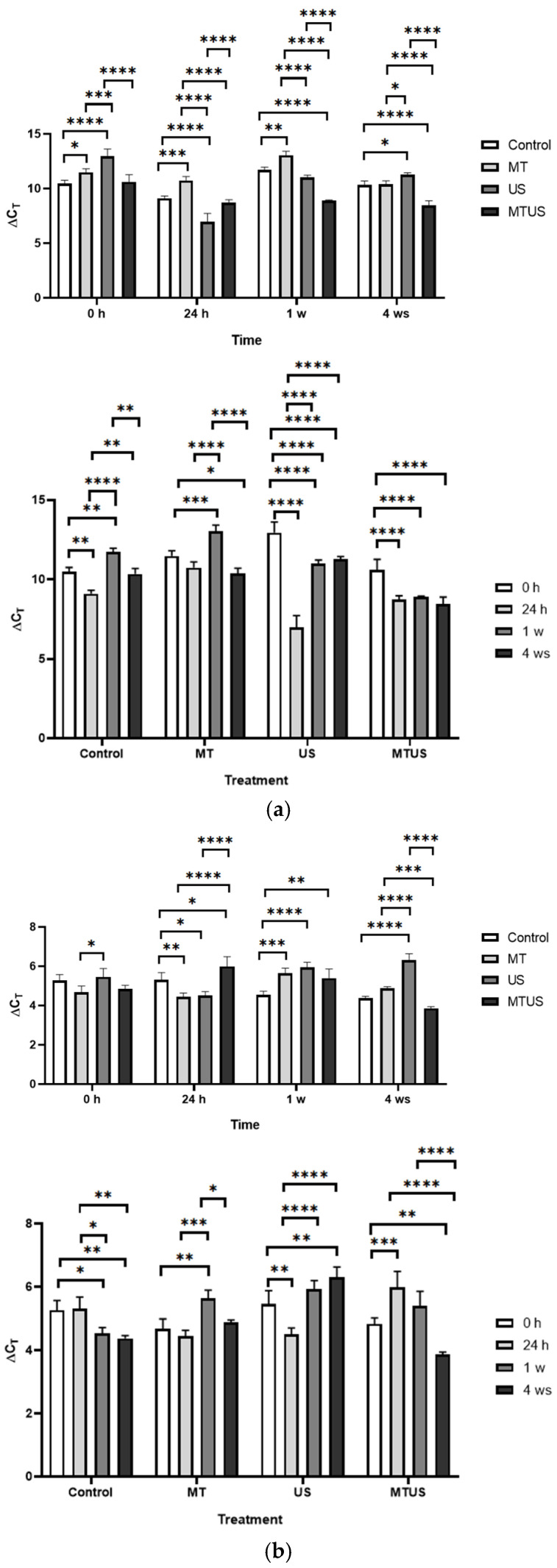
The expression levels of AANAT (**a**) and SNAT (**b**). Abbreviations: melatonin-containing medium (MT), ultrasound treatment (US) and combined treatment (MTUS). Statistics: no difference *p* > 0.05; * *p* ≤ 0.05; ** *p* ≤ 0.01; *** *p* ≤ 0.001; **** *p* ≤ 0.0001.

In the case of *AANAT* (Figure 17a), at 0 h, both MT and US samples showed increased levels, but at 24 h and at week 1, this was only typical for MT. The MTUS value was always lower than the other values, except for at 24 h, and this did not change even at week 4, by which time the other values were already normalized.

In the case of *SNAT* (Figure 17b), the difference was still minimal at 0 h, but at 24 h, the MT and US values were significantly lower than in the control and MTUS. By the first week, the increase had already started in both the MT and US samples and reached the level of MTUS. At this time, the control was significantly lower. By week 4, the measured values also began to normalize in this case, and only the *SNAT* content of the US sample remained significantly higher compared to the others at the same time.

#### 3.6.5. ASMT and COMT Genes

**Figure 18:**
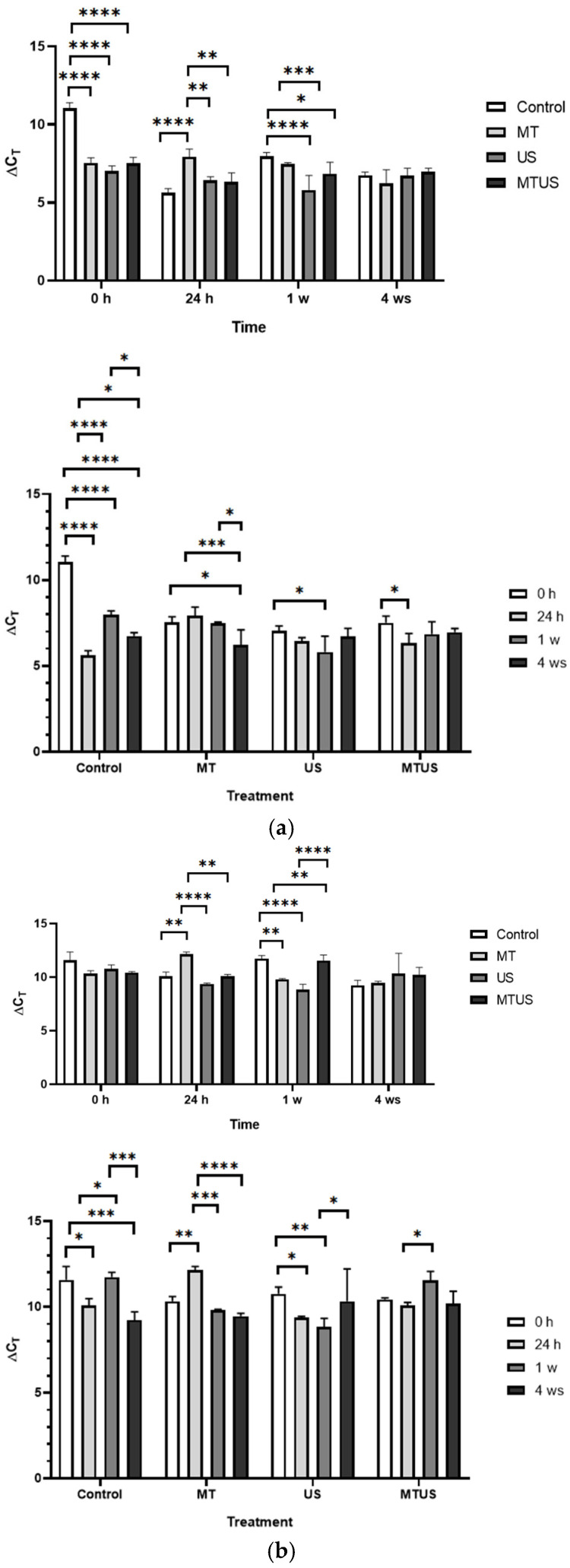
The expression levels of ASMT (**a**) and COMT (**b**). Abbreviations: melatonin-containing medium (MT), ultrasound treatment (US) and combined treatment (MTUS). Statistics: no difference *p* > 0.05; * *p* ≤ 0.05; ** *p* ≤ 0.01; *** *p* ≤ 0.001; **** *p* ≤ 0.0001.

In the case of *ASMT* (Figure 18a), at the initial time point, the MT and US treatments showed increased expression intensity compared to the control and only MTUS was significantly lower; by 24 h, only MT was higher compared to others at the same time. By the end of the 1st week, the MT value was already the same as the control while the other two treatments were the same but significantly lower than the control and MT. By the 4th week, the effect of the treatments was also observed to fade in this case, as by this time no significant differences were found.

At 0 h, the *COMT* results were not significantly different compared to the control (Figure 18b), but US and MTUS were significantly higher compared to the MT samples. At 24 h, the values measured for the treatments did not change significantly, but the value of the control increased so much that the value of the MT and MTUS samples became significantly lower. By the end of the 1st week, the control value had decreased to a level similar to the starting value, while the three treated samples showed an intensive increase. By the 4th week, the control value and the US value did not change significantly; however, the MT and MTUS values decreased so much that they were no longer significantly higher than the control. Thus, the treatments began to have their effect by the 1st week, when the amount of *COMT* measurable in the treated samples increased, but this effect remained permanent only in the case of US.

#### 3.6.6. TAA Gene

**Figure 19:**
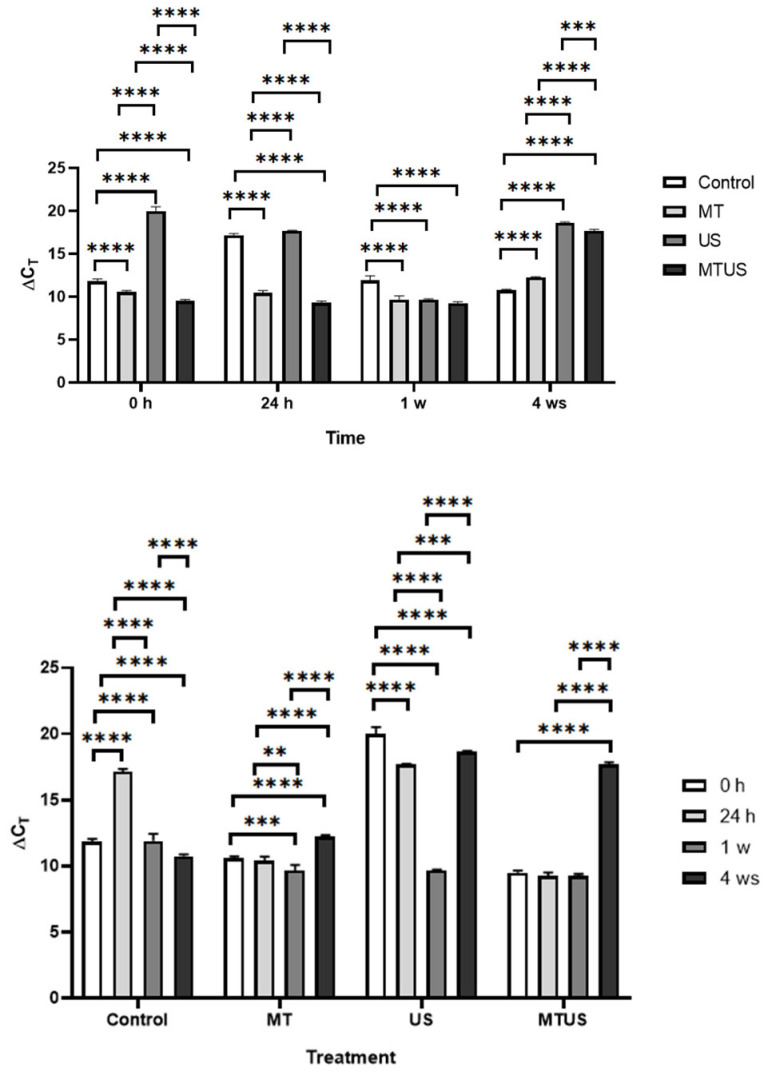
TAA expression levels. Abbreviations: melatonin-containing medium (MT), ultrasound treatment (US) and combined treatment (MTUS). Statistics: no difference *p* > 0.05; ** *p* ≤ 0.01; *** *p* ≤ 0.001; **** *p* ≤ 0.0001.

Among the enzymes examined, only TAA was more closely related to IAA synthesis (Figure 5). Figure 19 shows that MT treatment typically suppressed the expression of the enzyme until week 4, when, as previously mentioned, the exogenous MT was depleted. At time 0 h, MT-treated samples showed a significant decrease compared to the control, while the US sample showed a significant increase. There was no change in the effect of the treatments compared to the previous time point, but for some reason, TAA expression in the control sample increased significantly by this time. In the first week, the values of the MT and MTUS samples remained unchanged, but the values of the control and US decreased significantly compared to the others at the same time. By the last week, we observed a significant increase in all treated samples compared to the control.

#### 3.6.7. Gene Expression Changes

**Figure 20:**
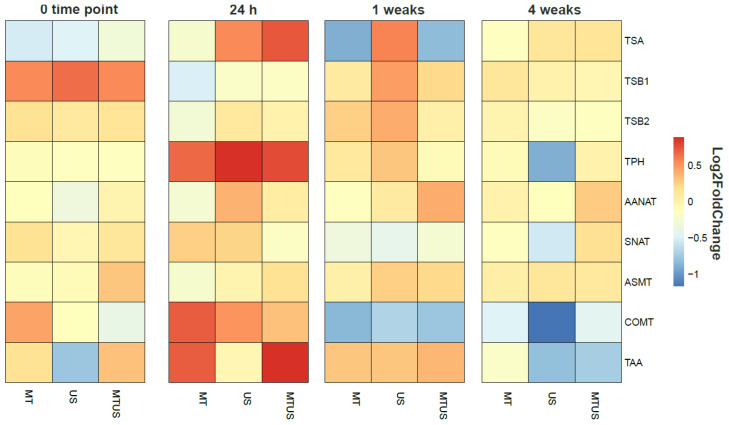
Display of gene expression levels in a heatmap. Abbreviations: melatonin-containing medium (MT), ultrasound treatment (US) and combined treatment (MTUS).

The summary figure (Figure 20) shows how the gene expression varied with treatment and over time compared to the control. It was observed that there was higher activity only at 24 h, as at this time, the genes were typically overexpressed, and in several cases, such as the genes responsible for the synthesis of the TPH, COMT and TAA enzymes, reached the red region. By week 1, the expression intensity was already decreasing, but this panel is still characterised by a darker orange colour, although several under-expressed genes were already present. By week 4, pale orange was the dominant colour and the number of blue regions increased further. No pattern typical of the treatments was seen in the figure.

## 4. Discussion

A review of the previous literature did not reveal any experimental description of the effect of exogenous MT in similar settings. As a consequence, we were only able to compare our measurement results with other works in parts. The primary, and perhaps most important, differences with our work are the method, dose and frequency of MT supply. In studies by other research groups, such as Jones (2025) [56], MT was applied to plants by spraying at a 20 mM concentration and repeated weekly for 5 weeks. In contrast, in our case, the medium contained MT at a 50 µM concentration, which was applied to the plant once, at the beginning of the subculture examined [56]. The next aspect is the interval of monitoring the effect of MT, as we did not encounter any experiments where the process was monitored for a longer period of time, i.e., with more sampling times. Typically, samples were collected only after the end of the experiment. Consequently, it was difficult to find adequate literature with which we could compare our results.

An important question in this experiment was whether and to what extent the plant would take up melatonin from the medium. In our experience, it is not typical for research teams to backtrack how much of the exogenous MT is actually taken up by the plant, nor how and over what time period the plant will utilize it [56].

We found that plant explants were able to take up large amount of MT from the culture medium, as the MT treated sample contained several times the value measured in the control plant. The determination of the concentration of MT was confirmed by ELISA and UHPLC-MS, and it was found that the concentration values of MT were significantly increased in the treated samples. The two measurements were compared and, as in the previously published literature [40,57], a significant difference between the techniques was observed. These differences can be attributed to several factors, such as extraction efficiency. Since we do not know the composition of the stabilizer/buffer used in the ELISA study, it could not be fully reproduced. Comparing the results, however, we can still conclude that although the quantified value is different, the pattern of the process is very similar. For example, both measurements confirmed that MT treatment increases MT concentrations in plant tissues and that US treatment decreases MT levels compared to the control. In the case of UHPLC, lower values were measured, most probably because in this case, only MT was selectively detected. This is supported by the retention time and the fragmentation spectrum (Figure 7). MT and its degradation products are structurally very similar, so it is possible to measure them by ELISA, as MT, and thereby obtain a higher value for MT. Although the concentration of MT decreased after US, the difference was not significant compared to the control.

The next aspect of the study was to investigate plant structure. Morphological data suggested that this extreme amount of exogenous MT was a major stress for the plant explants; much greater than the applied abiotic stress factor, the US. This resulted in poor plant growth in the initial period, but as MT levels within the plantlet normalized by week 4, the stress was removed and greater amounts of biomass were produced compared to the control. The positive effects of exogenous melatonin on biomass have been demonstrated in various plants, as reported in a review by Iqbal et al. (2022) [58]. In addition, our results support the finding of Wang et al. (2013) that exogenous melatonin has a positive effect on plant chlorophyll content [58,59].

The degradation products of exogenous MT in plants are receiving increasing attention, as not only MT itself but also its metabolites are known to have, for example, strong antioxidant effects. When examining the degradation products of MT, we found that two major degradation products appeared, AMK and 6HM. This was interesting because some literature suggested that 6HM was a form that only occurs in animals [29]. In contrast, we detected the presence of AMK and 6HM in our plant samples, identified by the retention time of the UHPLC-MS spectrum and the fragmentation products of the molecule.

**Figure 21:**
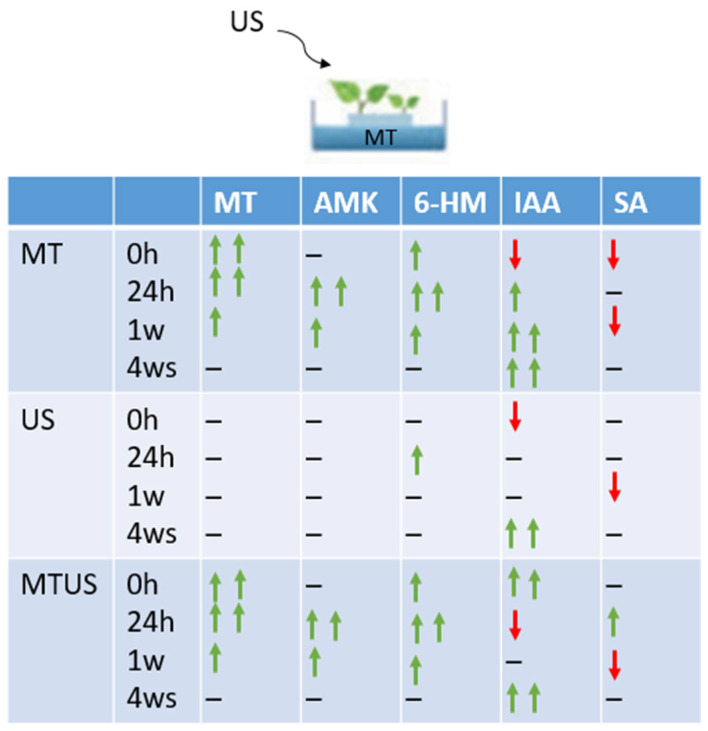
Summary figure of the measured analytical parameters. Abbreviations: Column: melatonin-containing medium (MT), ultrasound treatment (US) and combined treatment. Row: Melatonin (MT), N-acetyl-5-methoxykynuramine (AMK), 6-hydroxymelatonin (6HM), 5-methoxytryptamine (5MTA), indole-3-acetic acid (IAA), salicylic acid (SA). Green arrow—increase; red arrow—decrease.

In the measured parameters, MT had a significant effect on the plants compared to the control, and changes in the measured parameters were eliminated by the 4th week, i.e., they returned to the values measured in the control samples (Figure 21). Both MT and its degradation products were excreted from the tissues by the end of the experimental period. In the case of ultrasound treatment, all three degradation products appeared at 24 h, but 6HM was the most abundant. In this case, the degradation products disappeared by the first week. Only in the US treatment was a significant increase observed by week 4.

MT and IAA are closely related in plants because they are derived from the same precursor (tryptophan) and often have overlapping or interacting hormonal effects. Zhang et al. (2022) found that low doses of melatonin stimulate IAA biosynthesis, but high concentrations (e.g., exogenous) reduce IAA levels [54]. Our results were similar in this case. We found that, despite the high amount of MT in the plants, IAA was produced in reduced amounts. In our study, the amount of MT and IAA showed an inverse correlation, and by the time MT and its degradation products disappeared from the system, the amount of IAA had increased significantly. The IAA content of the plant decreased as a result of ultrasound and then increased steadily until the fourth week, but the difference was significantly greater at the last time point compared to the control.

SA was present in the plant in both free and bound forms and was mobilized when needed. This typically occurs in response to some pathogens [60]. As no infection occurred in this case, no drastic differences were observed compared to the control.

In addition, the expression of genes related to the enzymes responsible for melatonin synthesis was examined. The question was whether the application of exogenous melatonin could affect the plants’ own (endogenous) melatonin synthesis via altering the expression intensity of related genes. We found that there was no pattern observed in response to the treatments, as no systematic increase or decrease in gene expression was seen in melatonin-treated samples. It was therefore concluded that the use of endogenous MT did not interfere with normal MT production in the seedling.

## 5. Conclusions

The ELISA method used to determine the concentration of MT gave significantly higher values, but the UHPLC-MS values are considered more accurate due to the selective detection of MT by UHPLC-MS, in contrast to ELISA, which can detect not only MT but also its degradation products as MT. It was concluded that the plant uptake of exogenous MT was high, as MT concentrations in MT treated plants were several times higher than those of the control. Based on plant morphology data, we found that MT-treated samples had higher biomass and higher chlorophyll concentrations by the end of the subculture (4th week). Melatonin degradation products, mainly AMK and 6HM, appeared in high amounts within 24 h. IAA was produced in reduced amounts under the influence of MT, but as the MT was depleted from the plant, its amount increased. No significant change was observed for SA. For the analytical parameters, we found that the effect of exogenous melatonin disappeared by week 4. In addition, no expression pattern change was found for the genes tested that corresponded to the treatments. In most cases, the US had no significant effect on the measured analytical parameters.

## Figures and Tables

**Table 1:** RT-qPCR primers for housekeeping and target/relevant genes of potato.

Gene	Forward Primer	Reverse Primer	Probe
*β-tubulin* LOC102577588	GAT GTT GTG CCA AAG GAT GT	AAC TTG TGG TCA ATG CGA GA	6-Fam-TGT GGC CAC CAT CAA GAC CAA-BHQ-1
*COMT* LOC102590869	AGT GGA TTT GTC ATG ATT GGA G	GTG GAA GTT GAT GTG TCT GG	6-Fam-CAA ATG GGA AAG TGA TAA TTG CGG A-BHQ-1
*SNAT* LOC102597415	GCT TCT CTT TAA CCC CAT CTC	GGA GAT TGA GAG TTT TGT GG	6-Fam-TGG TGA TTG GAA TGG GAA TGG G-BHQ-1
*TSB1* LOC110769205	TGC TAA TGA CGA GGA CTT TCA G	TCT TCA GAT AAA CGT GAG GCC	
*TSB2* LOC110748759	TTT GGT CTT GAG TGT GAG GTG	CGG TTA AGT TAG ATG GAG ATG GG	
*TAA* LOC110773598	CCT GTA CCT TTA TGC TTC CTC G	TCT GCC CAA TTA CCC CAA AC	
*TPH* LOC4351945	CCA ACA CAT CTC CAT TGC TG	TGG TGA GCT CCA TAT CGT C	
*AANAT* LOC110757692	GGA GTA CAG GAA GAC GAA GAG	CGT CCC ATA TAG TTG CGT TG	
*ASMT* LOC4346795	GGT AGA GGA TAG CAG TGT CG	GCA CTT GAT GTC AGG TG	
*TSA* LOC110763488	GCA GTT GGT TTT GGC ATC TC	TGC TAG TTC CTT CAA CCC TTC	

Abbreviations: *COMT*, *caffeic acid O-methyltransferase*; *SNAT*, *serotonin N-acetyltransferase*; *TSB*, *tryptophan synthase B*; *TAA*, *tryptophan aminotransferase*; *TPH*, *tryptophan hydroxylase*; *AANAT*, *arylalkylamine N-acetyltransferase*; *ASMT*, *N-acetylserotonin methyltransferase*; *TSA*, *tryptophan synthase A*.

**Table 2:** Morphological parameters of 1-week-old in vitro seedlings after cultivation on melatonin-containing medium (MT), ultrasound treatment (US) and combined treatment (MTUS).

	Treatments			
	Control	US	MT	MTUS
Shoot number/explant (n = 150/treatment)	1.2 ± 0.049 a	1.3 ± 0.54 a	1.0 ± 0.026 b	1.3 ± 0.057 a
Shoot length (mm) (n = 150/treatment)	23.07 ± 0.61 a	22.15 ± 0.73 a	12.43 ± 0.75 c	17.33 ± 0.90 b
Number of nodes/plantlet (n = 150/treatment)	4.22 ± 0.12 b	4.17 ± 0.12 b	3.44 ± 0.19 c	5.07 ± 0.18 a
Shoot weight (mg/vessel) (n = 5/treatment)	1004.2 ± 125.8 ab	1462.5 ± 180.9 a	623.6 ± 78.2 b	908.4 ± 163.7 ab
Root length (mm) (n = 150/treatment)	19.01 ± 0.88 a	20.19 ± 0.92 a	9.66 ± 0.56 c	16.29 ± 0.93 b
Root weight (mg/vessel) (n = 5/treatment)	515.2 ± 31.1 a	617.3 ± 73.5 a	136.9 ± 39.6 b	184.3 ± 30.0 b

Mean values (±standard error) followed by different letters in each row indicate significantly (*p* ≤ 0.001, at the shoot weight *p* ≤ 0.05) different values between treatments, according to ANOVA followed by Tukey’s test.

**Table 3:** Morphological parameters of 4-week-old in vitro seedlings after cultivation on melatonin-containing medium (MT), ultrasound treatment (US) and combined treatment (MTUS).

	Treatments			
	Control	US	MT	MTUS
Shoot number/explant (n = 150/treatment)	1.2 ± 0.024 b	1.1 ± 0.023 b	1.4 ± 0.040 a	1.2 ± 0.025 b
Shoot length (mm) (n = 150/treatment)	45.41 ± 0.825 a	39.43 ± 0.703 b	39.43 ± 0.865 b	36.33 ± 0.728 c
Number of nodes/plantlet (n = 150/treatment)	6.86 ± 0.087 b	6.91 ± 0.091 b	7.58 ± 0.114 a	6.17 ± 0.090 c
Shoot weight (mg/vessel) (n = 5/treatment)	2448.0 ± 174.8 b	3255.5 ± 83.19 a	2758.5 ± 145.0 ab	3000.0 ± 115.1 a
Root length (mm) (n = 150/treatment)	77.05 ± 1.21 b	85.91 ± 1.36 a	72.79 ± 1.85 b	85.80 ± 1.88 a
Root weight (mg/vessel) (n = 5/treatment)	2611.1 ± 262.6 a	2650.2 ± 194.1 a	1582.5 ± 113.1 b	1485.5 ± 118.2 b
chl a content (µg/g FW) (n = 6/treatment)	655.5 ± 65.2 b	610.8 ± 46.1 b	1944.3 ± 221.1 a	1688.2 ± 59.01 a
chl b content (µg/g FW) (n = 6/treatment)	222.3 ± 19.1 b	204.0 ± 13.1 b	634.1 ± 48.9 a	560.1 ± 13.37 a
chl a + chl b content (µg/g FW)	879.4 ± 83.9 b	816.2 ± 58.7 b	2582.9 ± 270.4 a	2252.3 ± 71.2 a
chl a/chl b	2.94 ± 0.08 a	2.99 ± 0.07 a	3.05 ± 0.11 a	3.01 ± 0.06 a

Mean values (±standard error) followed by different letters in each row indicate significantly (*p* ≤ 0.001, at the shoot weight *p* ≤ 0.05) different values between treatments, according to ANOVA followed by Tukey’s test.

## Data Availability

The data sets generated and analysed during the current study are available from the corresponding author upon reasonable request.

## Abbreviations

The following abbreviations are used in this manuscript:

5MTA	5-Methoxytryptamine
6HM	6-Hydroxymelatonin
AANAT	Arylalkylamine N-acetyltransferase
AFMK	N-Acetyl-N-formyl-5-methoxykynuramine
AMK	N-Acetyl-5-methoxykynuramine
ASDAC	N-Acetylserotonin deacetylase
ASMT	N-Acetylserotonin methyltransferase
Chl a	Chlorophyll a
Chl b	Chlorophyll b
COMT	Caffeic acid O-methyltransferase
ELISA	Enzyme-linked immunosorbent assay
HPLC	High performance liquid chromatography
IAA	Indole-3-acetic acid
IDO	Indoleamine-2,3-dioxygenase
M2H	Melatonin-2-hydroxylase
M3H	Melatonin-3-hydroxylase
MT	Melatonin
MTUS	Melatonin and ultrasound treatment
RNS	Reactive nitrogen species
ROS	Reactive oxygen species
SA	Salicylic acid
SNAT	Serotonin N-acetyltransferase
T5H	Tryptamine 5-hydroxylase
TAA	Tryptophan aminotransferase
TDC	Tryptophan decarboxylase
TPH	Tryptophan hydroxylase
TSA	Tryptophan synthase A
TSB1	Tryptophan synthase B1
TSB2	Tryptophan synthase B2
US	Ultrasound treatment
